# Biodiversity surveys of grassland and coastal habitats in 2021 as a documentation of pre-war status in southern Ukraine

**DOI:** 10.3897/BDJ.11.e99605

**Published:** 2023-03-06

**Authors:** Nadiia Skobel, Dariia Borovyk, Denys Vynokurov, Ivan Moysiyenko, Andriy Babytskiy, Iryna Bednarska, Olesia Bezsmertna, Olha Chusova, Polina Dayneko, Jürgen Dengler, Riccardo Guarino, Kateryna Kalashnik, Alexander Khodosovtsev, Vitalii Kolomiychuk, Oksana Kucher, Anna Kuzemko, Viktor Shapoval, Olha Umanets, Natalia Zagorodniuk, Maryna Zakharova, Iwona Dembicz

**Affiliations:** 1 Kherson State University, Kherson, Ukraine Kherson State University Kherson Ukraine; 2 University of Warsaw, Warsaw, Poland University of Warsaw Warsaw Poland; 3 M.G. Kholodny Institute of Botany, NAS of Ukraine, Kyiv, Ukraine M.G. Kholodny Institute of Botany, NAS of Ukraine Kyiv Ukraine; 4 Masaryk University, Brno, Czech Republic Masaryk University Brno Czech Republic; 5 University of the Basque Country UPV/EHU, Bilbao, Spain University of the Basque Country UPV/EHU Bilbao Spain; 6 I.I. Schmalhausen Institute of Zoology NAS of Ukraine, Kyiv, Ukraine I.I. Schmalhausen Institute of Zoology NAS of Ukraine Kyiv Ukraine; 7 Institute of Ecology of the Carpathians, NAS of Ukraine, Lviv, Ukraine Institute of Ecology of the Carpathians, NAS of Ukraine Lviv Ukraine; 8 Educational and scientific center “Institute of biology and medicine”, Taras Shevchenko National University of Kyiv, Kyiv, Ukraine Educational and scientific center “Institute of biology and medicine”, Taras Shevchenko National University of Kyiv Kyiv Ukraine; 9 Zurich University of Applied Sciences (ZHAW), Wädenswil, Switzerland Zurich University of Applied Sciences (ZHAW) Wädenswil Switzerland; 10 Insitute of Botany of Slovak Academy of Sciences, Bratislava, Slovakia Insitute of Botany of Slovak Academy of Sciences Bratislava Slovakia; 11 Bayreuth Center of Ecology and Environmental Research (BayCEER), Bayreuth, Germany Bayreuth Center of Ecology and Environmental Research (BayCEER) Bayreuth Germany; 12 University of Palermo, Palermo, Italy University of Palermo Palermo Italy; 13 Institute of Marine Biology of the NAS of Ukraine, Odesa, Ukraine Institute of Marine Biology of the NAS of Ukraine Odesa Ukraine; 14 O.V. Fomin Botanical Garden Taras Shevchenko National University of Kyiv, Kyiv, Ukraine O.V. Fomin Botanical Garden Taras Shevchenko National University of Kyiv Kyiv Ukraine; 15 Falz-Fein Biosphere Reserve "Askania-Nova" NAAS of Ukraine, Askania-Nova, Ukraine Falz-Fein Biosphere Reserve "Askania-Nova" NAAS of Ukraine Askania-Nova Ukraine; 16 Black Sea Biosphere Reserve, NAS of Ukraine, Hola Prystan, Ukraine Black Sea Biosphere Reserve, NAS of Ukraine Hola Prystan Ukraine

**Keywords:** bryophytes, dry grasslands, flora, lichens, occurrence data, sampling-event data, steppe, vascular plants

## Abstract

**Background:**

This paper presents two sampling-event datasets with occurrences of vascular plants, bryophytes and lichens collected in May-June 2021 in southern Ukraine. We aimed to collect high-quality biodiversity data in an understudied region and contribute it to international databases and networks. The study was carried out during the 15th Eurasian Dry Grassland Group (EDGG) Field Workshop in southern Ukraine and the Dark Diversity Network (DarkDivNet) sampling in the Kamianska Sich National Nature Park. By chance, these datasets were collected shortly before the major escalation of the Russian invasion in Ukraine. Surveyed areas in Kherson and Mykolaiv Regions, including established monitoring plots, were severely affected by military actions in 2022. Therefore, collected data are of significant value in the context of biodiversity documentation. The knowledge about the biodiversity of this area will help to assess the environmental impact of the war and plan restoration of the damaged or destroyed habitats. The first preliminary analysis of collected data demonstrates the biodiversity richness and conservation value of studied grassland habitats.

**New information:**

We provide sampling-event datasets with 7467 occurrences, which represent 708 taxa (vascular plants, bryophytes and lichens) collected in 275 vegetation relevés. Amongst them, vascular plants are represented by 6665 occurrences (610 taxa), lichens - 420 (46) and bryophytes - 381 (51). Several new species were reported for the first time at the national or regional level. In particular, one vascular plant species (*Torilispseudonodosa*) and two lichen species (*Cladoniaconista*, *Endocarponloscosii*) were new to Ukraine. One vascular plant (*Stipatirsa*), two species of bryophytes (*Rhynchostegiummegapolitanum*, *Ptychostomumtorquescens*) and three species of lichens (*Cladoniacervicornis*, *C.symphycarpa*, *Involucropyreniumbreussi*) were recorded for the first time for the Kherson Region. Additionally, these datasets contain occurrences of taxa with narrow distribution, specialists of rare habitat types and, therefore, represented by a low number of occurrences in relevant biodiversity databases and particularly in GBIF. This publication highlights the diversity of natural vegetation and its flora in southern Ukraine and raises conservation concerns.

## Introduction

Studies of biodiversity and its patterns are central topics of ecology and biogeography. Besides, they also play a crucial role in sustainable development, nature conservation and ecosystem restoration (Gaston 2000, Whittaker et al. 2005). Natural and semi-natural grasslands of the Palaearctic biogeographic realm are of special interest for ecological research since they host high biodiversity (Biurrun et al. 2021). Moreover, grasslands and coastal habitats are amongst the most threatened ecosystems in Europe due to habitat fragmentation, plant invasion, abandonment, misuse, overexploitation or physical destruction (Beck and Airoldi 2010, Habel et al. 2013, Deák et al. 2016, Czarniecka-Wiera et al. 2019). In Ukraine, a strong decline in these ecosystems has been observed during the last two centuries. For instance, steppe vegetation historically covered approximately 40% of the total territory of the country, but today, steppe remnants exist only in 1% of this territory (Burkovskyi et al. 2013, Dembicz et al. 2016). However, preserved until now, areas of grassland vegetation maintain high biodiversity, including numerous rare and endemic plant species in Ukraine (Chusova et al. 2022, Kuzemko et al. 2022).

Studies of local biodiversity "blankspots" and under-represented taxa are important to supplement our knowledge about the diversity of ecosystems across the globe and to support their appropriate management and conservation. Ukrainian grasslands, with their known high diversity, are still quite understudied compared to other grassland regions (Didukh et al. 2020, Kuzemko et al. 2022, Moysiyenko et al. 2022). To address this issue, we performed two field studies in southern Ukraine in 2021: the 15th EDGG Field Workshop and the DarkDivNet sampling in the Kamianska Sich National Nature Park. Both activities, based on the collaboration of an international team of researchers, resulted in the collection of high-quality biodiversity data on vascular plants, bryophytes and lichens.

## General description

### Purpose

**Figure 1.**
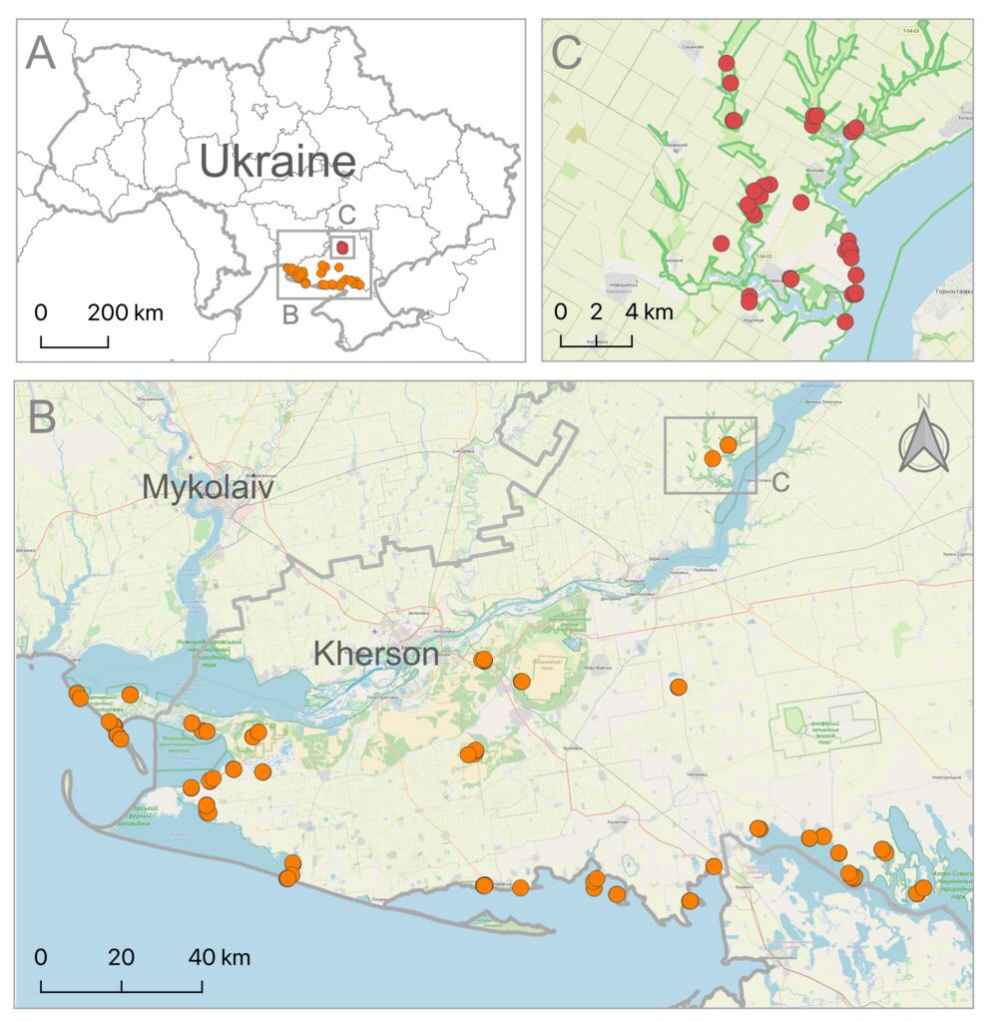
The map of the study region with sampling localities: **A** map of Ukraine with study area and sampling localities; **B** sampling localities of the 15th Eurasian Dry Grassland Group (EDGG) Field Workshop in southern Ukraine; **C** sampling localities of the Dark Diversity Network (DarkDivNet) in the Kamianska Sich National Nature Park. The base map was created using OpenStreetMap data (https://www.openstreetmap.org, as on December 2022).

We collected high-quality biodiversity data in the understudied region to contribute it to international databases and networks (GrassPlot, DarkDivNet). We publish collected data about species occurrences in surveyed sampling locations as a part of the baseline inventory of biodiversity and its pre-war status in the study area (Fig. 1).

## Project description

### Title

Northern Eurasia 2022

### Personnel

N-Eurasia-2022

### Funding

These datasets were created during the data mobilisation from across Northern Eurasia, initiated by GBIF in collaboration with the Finnish Biodiversity Information Facility (FinBIF) and Pensoft. The fieldwork for data collection was organised and funded by the Eurasian Dry Grassland Group (EDGG Field Workshop) and the Department of Botany of Kherson State University (EDGG Field Workshop, DarkDivNet sampling). N.Z. conducted the identification of bryophytes with the support of the EDGG Small Grant Program (https://edgg.org/supportUkraine). The data processing and publication were funded by “Documenting of phytodiversity of nature protected areas of Lower Dnipro Region” (CPEA-LT-2017/10049) and NCN scholarship programme for Ukrainian students and young researchers in collaboration with the University of Warsaw (nr 2021/01/4/NZ9/00078) (N.S.). The work of D.B. (previously as Dariia Shyriaieva) was supported by the Visegrad Fund (Scholarship #52010644) and the Scholarship programme of Masaryk University. The work of I.M. was conducted with the support of the U.S. Government Project No. 21GR3259 "Together for Environmental Democracy, Justice, and the Rule of Law in Ukraine" (TEDJusticeROL) (Subproject "Security of Protected Areas in the Context of Military Conflict and Occupation").

## Sampling methods

### Study extent

The study area is situated in the central-southern part of the Black Sea Lowland in the southern part of the steppe zone of Ukraine (Bohn and Neuhäusl 2004), in the Kherson and Mykolaiv administrative regions, within two geobotanical regions according to the national vegetation classification system (Barbarych 1977), namely sagebrush-fescue-steppes (or desert steppes) and fescue-feathergrass-steppes (or bunchgrass steppes). The climate of the study area is characterised by hot summers with a long dry period and short mild winters with little snow cover (Marynych and Shyshchenko 2005, Fig. 2). In the desert steppe zone, soils are mainly dark kastanozems, in combination with saline soils; in the belt of bunchgrass steppes, they are southern chernozems with low amounts of humus and alluvial deposits of the sand terraces of new and older riverbeds of the Dnipro River (Skliar and Hil’chenko 1969, Sudnik-Wójcikowska and Moysiyenko 2012). Mean annual precipitation ranges from 300 mm in the south to 400 mm in the north. Mean temperatures of the warmest month of July are 23–24°C (maximum 39°C); mean temperatures of the coldest month of January range from -1 to -4℃ (minimum -31°C) (Marynych and Shyshchenko 2005).

During the sampling, we surveyed the following protected areas: Black Sea Biosphere Reserve, Azov-Syvash National Nature Park, Dzharylgach National Nature Park, Biloberezhzhia Sviatoslava National Nature Park, Oleshkivski Pisky National Nature Park and Kamianska Sich National Nature Park (Moysiyenko et al. 2020), as well as territories outside of protected areas.

### Sampling description

To obtain the dataset “Records of vascular plants, bryophytes and lichens from the 15th EDGG Field Workshop Grasslands and coastal habitats of Southern Ukraine” (Moysiyenko et al. 2023b), we sampled 239 plots. Amongst them, 175 plots had an area of 10 m^2^; in some cases, these plots had additional larger grain sizes of 100 m^2^ (49 plots) and 1000 m^2^ (15 plots). The plots of larger size (100 and 1000 m^2^), which include smaller plots, are marked as parent events in the Event Core. Sampling locations were chosen in order to cover different types of natural and semi-natural open grassland habitats within the study area. In each plot, we recorded occurrences of vascular plants (shoot presence), terricolous soil-growing bryophytes and lichens. For several plots, epiphytic lichens on woody plants were also registered. For each 10-m^2^ plot, the following environmental and structural parameters were recorded: cover of vegetation layers (total vegetation cover, shrubs, herbs, cryptogams), cover of litter, cover of rocks, aspect, inclination, shrub layer height and herb layer height. The complete relevés with 7-8 nested-plot biodiversity series collected during the 15th EDGG Field Workshop following the EDGG methodology (Dengler et al. 2016, Dengler et al. 2021) are available in the GrassPlot database (Dengler et al. 2018).

The second dataset, “Records of vascular plants from DarkDivNet sampling in Kamianska Sich National Nature Park (site D194)” (Moysiyenko et al. 2023a), consists of occurrences of vascular plants registered in the plots which were established during the DarkDivNet sampling (Lewis et al. 2015). Within an area with a radius of 10 km, we established two permanent core plots and 34 accompanying subplots (only occurrences of 32 of them are presented in the current dataset, as indicated in Study Extent). Two permanent plots represented natural (unmanaged bunchgrass steppe) and semi-natural (intensively grazed pasture, previously ploughed) grassland habitats. Each permanent plot had a nested structure of two scales: a core plot (100 m^2^) within a surrounding plot (2500 m^2^), which is indicated as a parent event in the dataset structure. We placed the subplots (100 m^2^ each) randomly in an area with a radius of 10 km around the centre of the study area, which was defined as the mid-point of two permanent plots. The subplots covered various types of relatively intact natural and semi-natural vegetation. Within each permanent core plot and subplot, we recorded all vascular plant species (shoot presence).

### Quality control

Some specimens of the vascular plants and almost all lichens and mosses were collected as herbarium specimens for determination and verification after the fieldwork. The collected materials were verified in the Laboratory of Plant Ecology and Environmental Protection (Department of Botany, KSU), the Herbarium of Kherson State University (KHER), Herbarium of the Institute of Ecology of Carpathians NAS of Ukraine (LWKS) and the Herbarium of Masaryk University in Brno (BRNU). After digitising the data, we harmonised the taxonomic information according to the following nomenclature sources: for vascular plants - Nomenclatural Checklist of vascular plants of Ukraine (Mosyakin and Fedoronchuk 1999), for bryophytes - An annotated checklist of bryophytes of Europe, Macaronesia and Cyprus (Hodgetts et al. 2020) and for lichens – The fourth checklist of Ukrainian Lichen-forming and lichenicolous fungi with analysis of current additions (Kondratyuk et al. 2021). Then we used GBIF Backbone Taxonomy (GBIF Secretariat 2022, GBIF species matching tool) for the taxonomic check and implemented minor corrections of species names regarding misprints and problematic taxa to avoid misinterpretation. We additionally checked and verified the header data of vegetation plots (Event Core and GBIF Relevé Extension, OccurrenceExtension) using OpenRefine, R (R Core Team 2022) and QGIS 3.22 (QGIS Development Team 2022) for quality control.

### Step description

The following steps were taken:

1. Site selection, field research.
2. Identification of herbarium specimens of vascular plants, lichens, bryophytes.
3. Digitalising the field data forms.
4. Harmonisation of the taxonomic information according to the nomenclature sources (Mosyakin and Fedoronchuk 1999, Hodgetts et al. 2020, Kondratyuk et al. 2021).
5. Data checking and cleaning (Hollister et al. 2021, OpenRefine 2022, QGIS Development Team 2022, R Core Team 2022).
6. Transformation of the dataset according to the Darwin Core standards (Wieczorek et al. 2012).
7. Taxonomic check using the GBIF Backbone Taxonomy (GBIF Secretariat 2022) and GBIF species matching tool and minor taxonomic corrections.
8. Final quality control (OpenRefine 2022).

## Geographic coverage

### Description

Kherson and Mykolaiv Regions, Ukraine.

## Taxonomic coverage

### Description

The dataset includes 708 taxa name records (vascular plants, bryophytes and lichens, details in Table 1), amongst them one taxon is determined to kingdom level, four - to classes, 44 - to genus, 642 - to species, four - to subspecies, one - to variety. We used the taxonRank *aggregate* for some taxonomically problematic taxa and unclear determinations: *Arenariaserpyllifolia* agg., *Asteramellu*s agg., *Cerastiumpumilum* agg., *Cetrariaaculeata* agg., *Consolidaregalis* agg., *Crataegusmonogyna* agg., *Festucavalesiaca* agg., *Lecaniellacyrtella* agg., *Placynthiellauliginosa* agg., *Tanacetummillefolium* agg., *Taraxacumofficinale* agg. and *Violatricolor* agg. Amongst all species records, the most common families of vascular plants are Asteraceae (1197 occurrence records), Poaceae (1184), Caryophyllaceae (617), Fabaceae (539), Brassicaceae (477). The most common taxa of vascular plants at the species level are *Poabulbosa* (116), *Holosteumumbellatum* (106), *Agropyronpectinatum* (90), *Bromussquarrosus* (80), *Lamiumamplexicaule* (80), *Seneciovernalis* (80), *Myosotismicrantha* (75), *Veronicaarvensis* (70) and *Arenariaserpyllifolia* s.l. (68). The most common taxa of bryophytes are *Syntrichiaruralis* (64), *Ceratodonpurpureus* (53), *Syntrichiaruraliformis* (22) and *Pterygoneurumovatum* (17). The most common taxa of lichens are *Cladoniafoliacea* (48), *Cladoniarangiformis* (44), *Cladoniafurcata* (39) and *Blennothalliacrispa* (23).

### Taxa included

**Table:** 

Rank	Scientific Name	
kingdom	Fungi	
kingdom	Plantae	
phylum	Ascomycota	
phylum	Bryophyta	
kingdom	Marchantiophyta	
kingdom	Tracheophyta	

## Temporal coverage

**Data range:** 2021-5-23 – 2021-6-24.

## Usage licence

### Usage licence

Open Data Commons Attribution License

## Data resources

### Data package title

Records of vascular plants, bryophytes and lichens from the 15th EDGG Field Workshop “Grasslands and coastal habitats of Southern Ukraine”.

### Resource link

https://www.gbif.org/uk/publisher/adc3d841-aefb-4d7c-9ab1-2156d614b27b

### Number of data sets

2

### Data set 1.

#### Data set name

Records of vascular plants, bryophytes and lichens from the 15th EDGG Field Workshop “Grasslands and coastal habitats of Southern Ukraine”.

#### Data format

Darwin Core Archive

#### Character set

UTF-8

#### Download URL

https://www.gbif.org/dataset/5eb9d316-3b09-414c-ad57-e57b206c354b

#### Data format version

csv

#### Description

The dataset includes a table with 44 fields in Darwin Core terms and 5917 records in it (Moysiyenko et al. 2023b).

**Data set 1.** 

Column label	Column description
eventID (Darwin Core Event, GBIF Relevé Extension, Darwin Core Occurrence Extension)	An identifier for the Event (based on dataset name and relevé number): EDGG2021_15FW_Ukraine_plotID.
parentEventID (Darwin Core Event)	An identifier of the broader Event that includes this and potentially other Events and does not allow duplicate species occurrences in nested samples.
sampleSizeValue (Darwin Core Event)	A numeric value for a measurement of a sample in a sampling event (10, 100, 1000).
sampleSizeUnit (Darwin Core Event)	The unit of measurement of the size of a sample in a sampling event (square metre).
samplingProtocol (Darwin Core Event)	The names of the methods or protocols used during an Event (Species Shoot Presence).
eventDate (Darwin Core Event)	The date when the Event occurred (from 2021-05-24 to 2021-06-03).
habitat (Darwin Core Event)	A category or description of the habitat in which the Event occurred.
country (Darwin Core Event)	The name of the country in which the Location occurs (Ukraine).
countryCode (Darwin Core Event)	The standard code for the country where the Location occurs (UA).
stateProvince (Darwin Core Event)	The name of the administrative region of Ukraine in which the Location occurs: Kherson and Mykolaiv Regions.
county (Darwin Core Event)	The name of the district, which is a smaller unit of administrative division than a region in the field stateProvince.
locality (Darwin Core Event)	The specific description of the place. Include the nearest village, protected area and relief position.
locationRemarks(Darwin Core Event)	Comments or notes about the Location, including field form ID and land use information.
decimalLatitude (Darwin Core Event)	The geographic latitude in decimal degrees.
decimalLongitude (Darwin Core Event)	The geographic longitude in decimal degrees.
geodeticDatum (Darwin Core Event)	The geodetic datum upon which the geographic coordinates are given (WGS84).
coordinateUncertaintyInMeters (Darwin Core Event)	The distance (in metres) from the given decimalLatitude and decimalLongitude describing the smallest circle containing the whole of the Location (30 m).
verbatimElevation (Darwin Core Event)	The original description of the elevation (altitude, usually above sea level) of the Location. Measurement with R package ‘evelator’.
georeferencedBy (Darwin Core Event)	Persons who georeferenced the position of an Event (relevé authors).
georeferenceProtocol (Darwin Core Event)	A description of the method used to determine coordinates (GPS).
coverTotalInPercentage (GBIF Relevé Extension)	The total cover (%) of all plants, rounded to the nearest hundredth.
coverShrubsInPercentage (GBIF Relevé Extension)	The cover (%) of shrubs, rounded to the nearest hundredth.
coverHerbsInPercentage (GBIF Relevé Extension)	The cover (%) of the herb layer, rounded to the nearest hundredth.
coverCryptogamsInPercentage (GBIF Relevé Extension)	The cover (%) of cryptogams, rounded to the nearest hundredth.
coverLitterInPercentage (GBIF Relevé Extension)	The cover (%) of litter, rounded to the nearest hundredth.
shrubLayerHeightInMeters (GBIF Relevé Extension)	The height in meters of the shrub layer, can be written in decimal notation.
herbLayerHeightInCentimeters (GBIF Relevé Extension)	The height in centimetres of the high herb layer, rounded to the nearest whole number.
coverRockInPercentage (GBIF Relevé Extension)	The cover (%) of bare rock, rounded to the nearest hundredth.
aspect (GBIF Relevé Extension)	The compass direction that the relevé site faces (Note: -1 flat area).
inclinationInDegrees (GBIF Relevé Extension)	The inclination of relevé site in degrees.
occurrenceID (Darwin Core Occurrence Extension)	An identifier of a particular occurrence, unique within this dataset. We used the species occurrence numbers. (EDGG2021_15FW_Ukraine_plotID_OccurrenceNumber).
scientificName (Darwin Core Occurrence Extension)	The original names according to the nomenclature sources (Mosyakin & Fedoronchuk 1999; Hodgetts et al. 2020; Kondratyuk et al. 2021), with minor corrections for spelling mistakes and problematic taxa.
verbatimIdentification (Darwin Core Occurrence Extension)	A string representing the taxonomic identification as it appeared in the original record.
identificationQualifier (Darwin Core Occurrence Extension)	A brief phrase or a standard term ("cf.", "aff.") to express the determiner's doubts about the Identification.
basisOfRecord (Darwin Core Occurrence Extension)	The method in which data were acquired (HumanObservation).
recordedBy (Darwin Core Occurrence Extension)	Persons who responsible for recording the original Occurrence. Authors of relevé.
identifiedBy (Darwin Core Occurrence Extension)	Persons who assigned the Taxon to the subject. Authors of plot or specialists in different groups of taxa: bryophytes – Natalia Zagorodniuk, lichens and lichenicolous fungi - Alexandr Khodosovtsev, genus *Cerastium* - Jiří Danihelka & Dariia Borovyk (previously as Dariia Shyriaieva), genus *Festuca* - Iryna Bednarska.
taxonRank (Darwin Core Occurrence Extension)	The taxonomic rank of the most specific name in the field scientificName.
kingdom (Darwin Core Occurrence Extension)	The full scientific name of the kingdom in which the taxon is classified.
phylum (Darwin Core Occurrence Extension)	The full scientific name of the phylum or division in which the taxon is classified.
class (Darwin Core Occurrence Extension)	The full scientific name of the class in which the taxon is classified.
order (Darwin Core Occurrence Extension)	The full scientific name of the order in which the taxon is classified.
family (Darwin Core Occurrence Extension)	The full scientific name of the family in which the taxon is classified.
genus (Darwin Core Occurrence Extension)	The full scientific name of the genus in which the taxon is classified.

### Data set 2.

#### Data set name

Records of vascular plants from DarkDivNet sampling in Kamianska Sich National Nature Park (site D194).

#### Data format

Darwin Core Archive

#### Character set

UTF-8

#### Download URL

https://www.gbif.org/dataset/24e6ef1c-8e52-48aa-a480-be658214f1b8

#### Data format version

csv

#### Description

The dataset includes a table with 41 fields in Darwin Core terms and 1970 records in it (Moysiyenko et al. 2023a).

**Data set 2.** 

Column label	Column description
eventID (Darwin Core Event, GBIF Relevé Extension, Darwin Core Occurrence Extension)	An identifier for the Event (based on dataset name and relevé number): (DarkDivNet2021_Ukraine_D194_plotID).
parentEventID (Darwin Core Event)	An identifier of the broader Event that includes this and potentially other Events and does not allow duplicate species occurrences in nested samples.
sampleSizeValue (Darwin Core Event)	A numeric value for a measurement of a sample in a sampling event (100, 250).
sampleSizeUnit (Darwin Core Event)	The unit of measurement of the size of a sample in a sampling event (square metre).
samplingProtocol (Darwin Core Event)	The names of the methods or protocols used during an Event (Species Shoot Presence).
eventDate (Darwin Core Event)	The date-time or interval during which an Event occurred (from 2021-05-23 to 2021-06-24).
habitat (Darwin Core Event)	A category or description of the habitat in which the Event occurred.
country (Darwin Core Event)	The name of the country in which the Location occurs (Ukraine).
countryCode (Darwin Core Event)	The standard code for the country where the Location occurs (UA).
stateProvince (Darwin Core Event)	The name of the administrative region of Ukraine in which the Location occurs (Kherson Region).
county (Darwin Core Event)	The full, unabbreviated name of the next smaller administrative unit than region (Beryslav District).
locality (Darwin Core Event)	The specific description of the place. Include the nearest village, protected area and relief position.
locationRemarks (Darwin Core Event)	Comments or notes about the Location, including field form ID and land use information.
decimalLatitude (Darwin Core Event)	The geographic latitude in decimal degrees.
decimalLongitude (Darwin Core Event)	The geographic longitude in decimal degrees.
geodeticDatum (Darwin Core Event)	The geodetic datum upon which the geographic coordinates are given (WGS84).
coordinateUncertaintyInMeters (Darwin Core Event)	The distance (in metres) from the given decimalLatitude and decimalLongitude describing the smallest circle containing the whole of the Location (30 m).
verbatimElevation (Darwin Core Event)	The original description of the elevation (altitude, usually above sea level) of the Location. Measurement with R package ‘evelator’.
georeferencedBy (Darwin Core Event)	Persons who georeferenced the position of an Event (relevé authors).
georeferenceProtocol (Darwin Core Event)	A description of the method used to determine coordinates (GPS).
coverTotalInPercentage (GBIF Relevé Extension)	The total cover (%) of all plants, rounded to the nearest hundredth.
coverShrubsInPercentage (GBIF Relevé Extension)	The cover (%) of shrubs, rounded to the nearest hundredth.
coverHerbsInPercentage (GBIF Relevé Extension)	The cover (%) of the herb layer, rounded to the nearest hundredth.
coverCryptogamsInPercentage (GBIF Relevé Extension)	The cover (%) of cryptogams, rounded to the nearest hundredth.
coverLitterInPercentage (GBIF Relevé Extension)	The cover (%) of litter, rounded to the nearest hundredth.
aspect (GBIF Relevé Extension)	The compass direction that the relevé site faces (Note: value -1 means flat area).
inclinationInDegrees (GBIF Relevé Extension)	The inclination of relevé site in degrees.
occurrenceID (Darwin Core Occurrence Extension)	An identifier of a particular occurrence, unique within this dataset. We used the species occurrence numbers (DarkDivNet2021_Ukraine_D194_plotID_OccurrenceNumber).
scientificName (Darwin Core Occurrence Extension)	The original names according to the nomenclature source (Mosyakin & Fedoronchuk 1999), with minor corrections for spelling mistakes and problematic taxa.
verbatimIdentification (Darwin Core Occurrence Extension)	A string representing the taxonomic identification as it appeared in the original record.
identificationQualifier (Darwin Core Occurrence Extension)	A brief phrase or a standard term ("cf.", "aff.") to express the determiner's doubts about the Identification.
basisOfRecord (Darwin Core Occurrence Extension)	The method in which data were acquired. (HumanObservation).
recordedBy (Darwin Core Occurrence Extension)	Persons who responsible for recording the original Occurrence. Authors of relevé.
identifiedBy (Darwin Core Occurrence Extension)	Persons who assigned the Taxon to the subject. Authors of plot, except of genus *Festuca* - Iryna Bednarska.
taxonRank (Darwin Core Occurrence Extension)	The taxonomic rank of the most specific name in the scientificName.
kingdom (Darwin Core Occurrence Extension)	The full scientific name of the kingdom in which the taxon is classified.
phylum (Darwin Core Occurrence Extension)	The full scientific name of the phylum or division in which the taxon is classified.
class (Darwin Core Occurrence Extension)	The full scientific name of the class in which the taxon is classified.
order (Darwin Core Occurrence Extension)	The full scientific name of the order in which the taxon is classified.
family (Darwin Core Occurrence Extension)	The full scientific name of the family in which the taxon is classified.
genus (Darwin Core Occurrence Extension)	The full scientific name of the genus in which the taxon is classified.

## Additional information

### Remarkable and protected species

**Figure 3.**
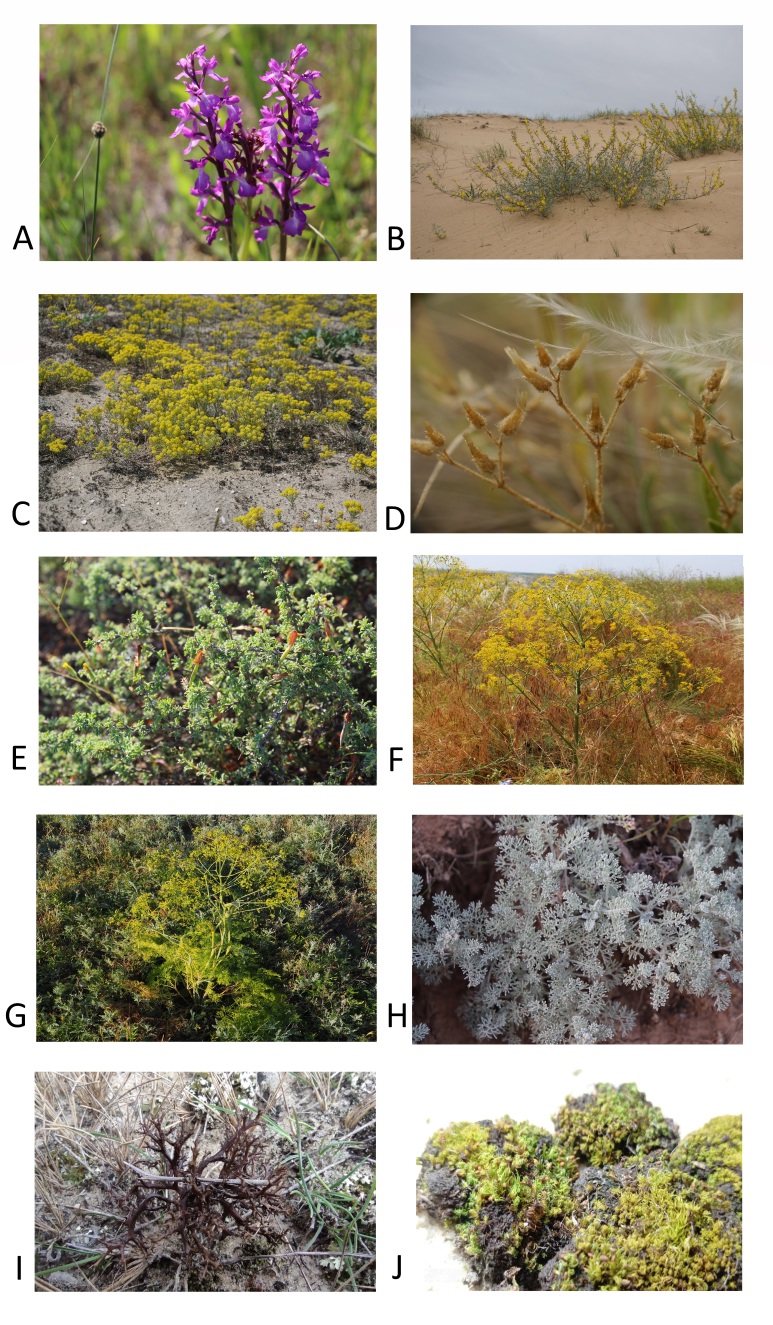
Rare and protected species of grasslands and coastal habitats in southern Ukraine: **A** – *Anacamptispalustris*; **B** - *Cytisusborysthenicus*; **C** – *Odontarrhenaborzaeana*; **D** - *Cerastiumschmalhauseni*; **E** – *Caraganascythica*; **F** - *Prangosodontalgica*; **G** - *Ferulacaspica*; H - *Artemisialerchiana*; **I** - *Cetrariaaculeata*; **J** - *Weissialoevieri* (photos A, E – O. Kucher, B, C, E - H - D. Borovyk; D - I. Dembicz; I - A. Khodosovtsev; J - N. Zagorodniuk).

A significant number of the sampled locations host occurrences of rare and protected species (Fig. 3). In particular, 30 species of vascular plants are protected by the Red Data Book of Ukraine (Didukh 2009): *Alliumregelianum*, *Alyssumborzaeanum*, *A.savranicum*, *Asparaguspallasii*, *Astragalusdasyanthus*, *A.odessanus*, *A.ponticus*, *A.reduncus*, *Bupleurumtenuissimum*, *Caraganascythica*, *Centaureabreviceps*, *Crambemaritima*, *Cymbochasmaborysthenicum*, *Elytrigiastipifolia*, *Genistascythica*, *Goniolimonrubellum*, *Limoniumtschurjukiense*, *Orchiscoriophora*, *O.picta*, *O.palustris*, *Stipaasperella*, *S.borysthenica*, *S.capillata*, *S.lessingiana*, *S.pennata*, *S.pulcherrima*, *S.tirsa*, *S.ucrainica*, *Thalictrumfoetidum* and *Tulipaschrenkii*. Two species of bryophytes (*Microbryumcurvicollum* and *Weissialevieri*) are listed in the Red Data Book of European bryophytes (ECCB 1995). In addition to species protected at the national and international levels, other rare species are also represented in the data, including the characteristic and endemic species of threatened habitat types, for example, *Artemisialerchiana*, *Cerastiumschmalhauseni*, *Cytisusborysthenicus* and *Prangosodontalgica* (Fig. 3).

## Figures and Tables

**Figure 2.**
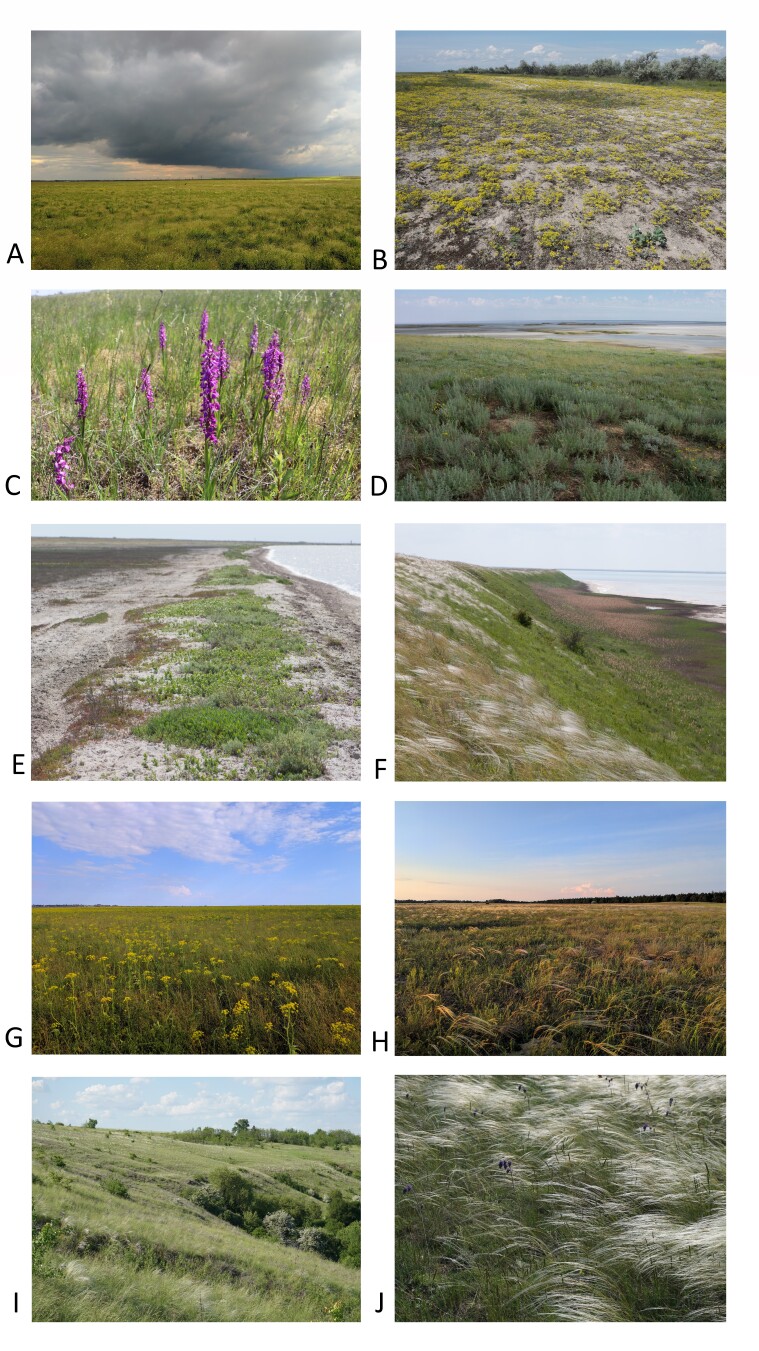
Grasslands and coastal habitats of southern Ukraine: **A** - steppe depression ("pody"); **B** - grey dune with Leymusracemosussubsp.sabulosus, *Odontarrhenaborzaeana* and *Carexcolchica*; **C** - sandy mesic grassland with *Scirpoidesholoschoenus* and *Anacamptispalustris*; **D** - desert steppes with *Artemisiataurica*, *A.lerchiana* and *Tanacetummillefolium* agg.; **E** – pioneer halo-nitrophilous vegetation on the coastline of the Yahorlyk Bay with *Cakileeuxina* and *Crambepontica*; **F** – dry bunchgrass steppes on the Syvash shore with *Stipalessingiana* and *Festucavalesiaca* aggr.; **G** – subsaline steppe, Potiivka site of the Black Sea Reserve with *Pastinacaclausii*, *Agropyronpectinatum*, *Halimioneverrucifera*; **H** – sandy steppe with dominated *Stipaborysthenica* in the Black Sea Reserve; **I** – steppe gully landscape in the Kamianska Sich National Park; **J** – community with *Stipalessingiana* and *Salvianutans* in Kamianska Sich National Park (photos A, G, H - D. Vynokurov, B, I, J - D. Borovyk, C - O. Kucher, D-F Dengler).

**Table 1.** Taxonomical distribution of Higher taxonomy. Notes: * VP - Vascular plants, B- Bryophyta, L - Lichens, All - all groups.

TaxonRank*	**Number of records (VP)**	**Number of records (B)**	**Number of records (L)**	**Number of records (All)**
**kingdom**	1	1	1	**2**
**phylum**	1	2	1	**4**
**class**	4	3	3	**10**
**order**	28	8	10	**46**
**family**	57	11	15	**83**
**genus**	280	30	31	**342**
